# Supplementary material for: Structural Basis for the Activation and Target Site Specificity of CDC7 Kinase
Source: Structure. 2020 Aug 4;28(8):954–962.e4. doi: 10.1016/j.str.2020.05.010 (PMC7416108; doi:10.1016/j.str.2020.05.010)
Supplement: Document S1. Figures S1 and S2 [file mmc1.pdf]

Structure, Volume *28*

Supplemental Information

Structural Basis for the Activation and Target Site Specificity of CDC7 Kinase

Samual D. Dick, Stefania Federico, Siobhan M. Hughes, Valerie E. Pye, Nicola O'Reilly, and Peter Cherepanov

Figure S1

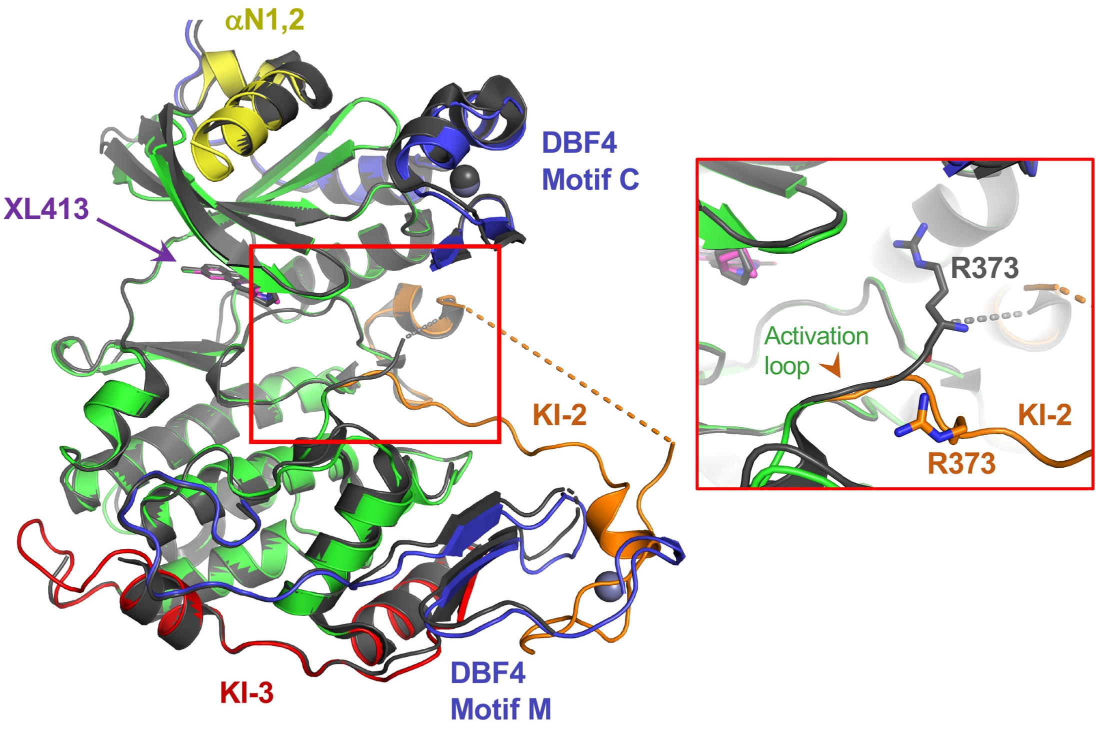

Figure S1. Comparison of the CDC7-DBF4 constructs with Δ228-359 (PDB ID 4F9C, grey) and Δ228-345 (this work, colored as in Fig. 2A) deletions in KI-2. Related to Figure 2A. Protein and the bound inhibitor (XL413) are depicted as cartoons and sticks, respectively. A zoomed view of the CDC7 activation segment region is shown in inset; note that the more extensive deletion in KI-2 leads to a disorder of the CDC7 activation segment.

Figure S2

A

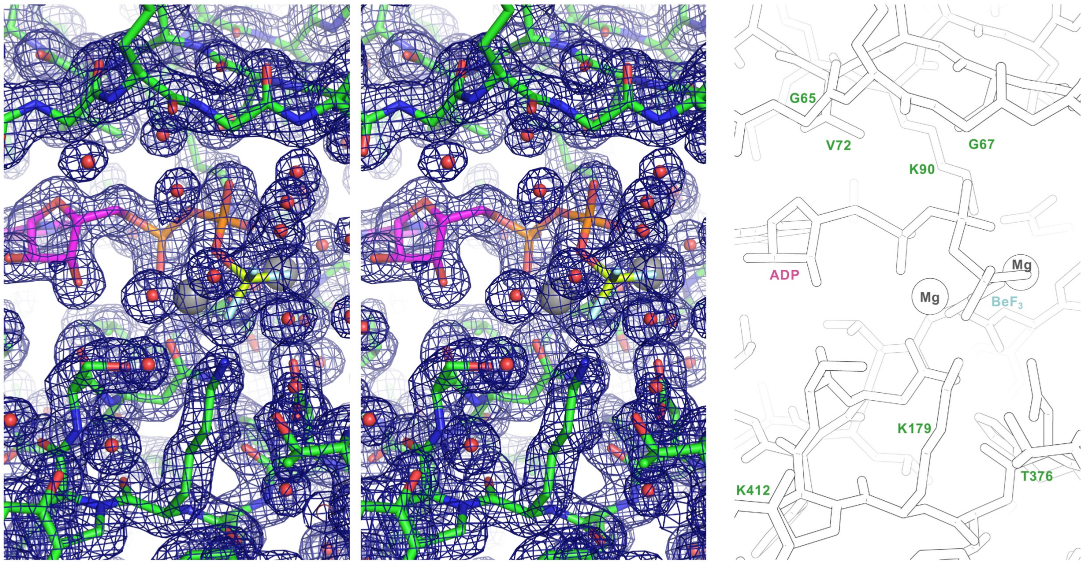

B

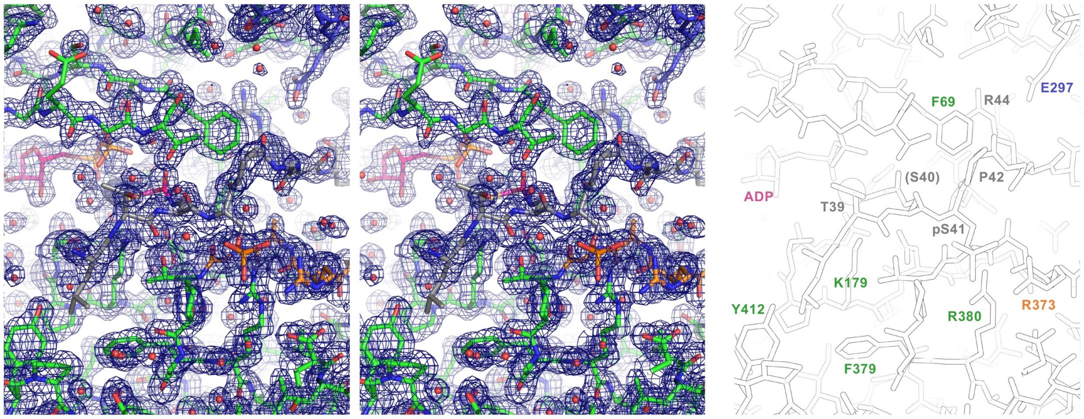

Figure S2. Stereo views of the final electron density maps of ADP-BeF3$^-$ (A) and bisubstrate (B) complexes. Related to Figures 2C and 3A. Weighted *2Fo-Fc* maps, contoured at 1 σ, are shown as blue mesh. Protein residues and ligands are depicted as sticks, water molecules as small red spheres and metal ions as grey spheres. Nucleotides and selected amino acid residues are indicated on the black and white images to the right.
