# Supplementary material for: Structural Basis for the Activation and Target Site Specificity of CDC7 Kinase
Source: Structure. 2020 Aug 4;28(8):954–962.e4. doi: 10.1016/j.str.2020.05.010 (PMC7416108; doi:10.1016/j.str.2020.05.010)
Supplement: Document S2. Article plus Supplemental Information [file mmc2.pdf]

Structure

Article

Structural Basis for the Activation and Target Site Specificity of CDC7 Kinase

Graphical Abstract

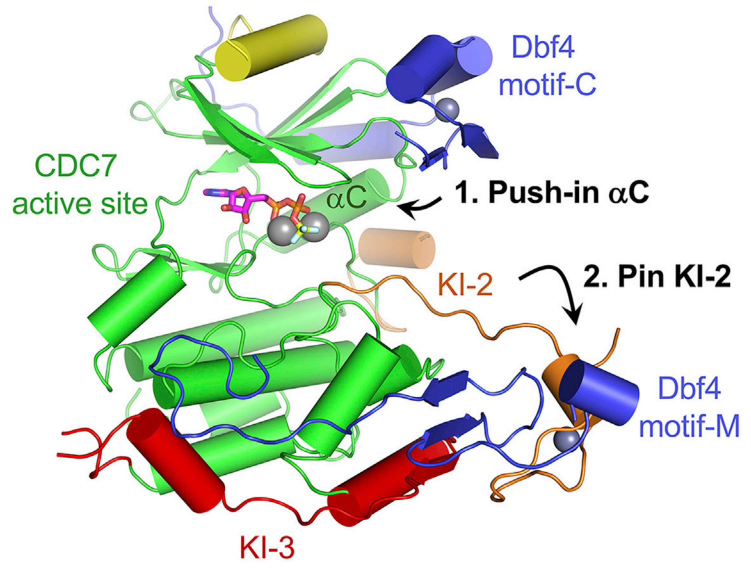

Highlights

- DBF4 activates CDC7 kinase via a two-step mechanism
- Zinc-finger domain in CDC7 KI2 interacts with DBF4 motif M
- Invariant CDC7 residues Arg373 and Arg380 engage P+1 substrate site

Authors

Samual D. Dick, Stefania Federico, Siobhan M. Hughes, Valerie E. Pye, Nicola O'Reilly, Peter Cherepanov

Correspondence

peter.cherepanov@crick.ac.uk

In Brief

CDC7 is a protein kinase that is essential for cell division. Using X-ray crystallography, Cherepanov and colleagues explain the two-step mechanism of CDC7 activation by its dedicated regulator protein DBF4. They also identify amino acid residues in CDC7 that are crucial for the recognition of its preferred substrates.

Dick et al., 2020, Structure *28*, 954–962
August 4, 2020 © 2020 The Authors. Published by Elsevier Ltd.
https://doi.org/10.1016/j.str.2020.05.010

CellPress
OPEN ACCESS

Structure

Article

Structural Basis for the Activation and Target Site Specificity of CDC7 Kinase

Samual D. Dick,[1,4] Stefania Federico,[2] Siobhan M. Hughes,[1] Valerie E. Pye,[1] Nicola O'Reilly,[2] and Peter Cherepanov[1,3,5,*]
[1]Chromatin Structure and Mobile DNA, The Francis Crick Institute, London NW1 1AT, UK
[2]Peptide Synthesis Laboratory, The Francis Crick Institute, London NW1 1AT, UK
[3]Division of Medicine, Imperial College London, London W2 1PG, UK
[4]Present address: British Heart Foundation, Greater London House, 180 Hampstead Road, London NW1 7AW, UK
[5]Lead Contact
*Correspondence: peter.cherepanov@crick.ac.uk
https://doi.org/10.1016/j.str.2020.05.010

SUMMARY

CDC7 is an essential Ser/Thr kinase that acts upon the replicative helicase throughout the S phase of the cell cycle and is activated by DBF4. Here, we present crystal structures of a highly active human CDC7-DBF4 construct. The structures reveal a zinc-finger domain at the end of the kinase insert 2 that pins the CDC7 activation loop to motif M of DBF4 and the C lobe of CDC7. These interactions lead to ordering of the substrate-binding platform and full opening of the kinase active site. In a co-crystal structure with a mimic of MCM2 Ser40 phosphorylation target, the invariant CDC7 residues Arg373 and Arg380 engage phospho-Ser41 at substrate P+1 position, explaining the selectivity of the S-phase kinase for Ser/Thr residues followed by a pre-phosphorylated or an acidic residue. Our results clarify the role of DBF4 in activation of CDC7 and elucidate the structural basis for recognition of its preferred substrates.

INTRODUCTION

CDC7 is a conserved Ser/Thr kinase, which, in addition to its essential role in activation of DNA replication origins (reviewed in Rossbach and Sclafani, 2016), is implicated in intra S-phase checkpoint (Costanzo et al., 2003; Duncker et al., 2002; Tsuji et al., 2008; Weinreich and Stillman, 1999; Yamada et al., 2013), DNA repair (Princz et al., 2017), mitotic exit (Miller et al., 2009; Mishra et al., 2016), meiosis (Buck et al., 1991; Katis et al., 2010; Lo et al., 2012; Matos et al., 2008; Murakami and Keeney, 2014; Ogino et al., 2006; Sasanuma et al., 2008; Wan et al., 2008), and chromosome cohesion (Natsume et al., 2013; Takahashi et al., 2008). Catalytically inert in isolation, CDC7 becomes functional in complex with Dumbbell forming factor 4 (DBF4), the levels of which fluctuate throughout the cell cycle (Ferreira et al., 2000; Jackson et al., 1993; Oshiro et al., 1999). The activity of CDC7 peaks at the G_1/S-phase transition and is maintained throughout S phase, during which the kinase fulfills its primary role of phosphorylating components of the replisome to facilitate loading of firing factors and subsequent DNA unwinding (Jackson et al., 1993; Oshiro et al., 1999). Well-characterized targets of phosphorylation by CDC7 are subunits of the heterohexameric replicative DNA helicase MCM2–MCM7. CDC7-mediated phosphorylation of MCM2 and MCM4 appears to be particularly important in humans and yeast, respectively (Masai et al., 2006; Montagnoli et al., 2006; Randell et al., 2010; Sheu and Stillman, 2010). In addition, CDC7 was implicated in phosphorylation of proteins involved in meiosis, DNA repair, and gene expression (Brandao et al., 2014; Hughes et al., 2010; Wan et al., 2008). While substrate recognition by CDC7 likely involves interactions with substrate regions distal to the phosphate acceptor residue (Sheu and Stillman, 2006), characterized phosphorylation sites share an acidic or a pre-phosphorylated residue at P+1 position (Cho et al., 2006; Hughes et al., 2010; Montagnoli et al., 2006; Princz et al., 2017).

CDC7 and DBF4 tend to be overexpressed in primary cancers and tumor cell lines, and their levels negatively correlate with patient prognosis (Bonte et al., 2008; Cheng et al., 2013; Clarke et al., 2009; Ghatalia et al., 2016; Hou et al., 2012; Jaafari-Ashkavandi et al., 2019; Kulkarni et al., 2009; Melling et al., 2015; Subramanian and Cohen, 2019; Zhuang et al., 2018). Selective inhibition of CDC7 suppresses proliferation of transformed cells through induction of S-phase delay and replication stress (Cheng et al., 2018; Im and Lee, 2008; Ito et al., 2012; Iwai et al., 2019; Montagnoli et al., 2004). Furthermore, CDC7 antagonists showed promise in combination with other cell-cycle inhibitors and anti-cancer therapeutics (Cao and Lu, 2019; Gad et al., 2019; O' Reilly et al., 2018). Selective inhibitors of CDC7 are being developed and trialed as anti-cancer therapeutics, with a number of candidate drugs undergoing clinical trials (Cheng et al., 2018; Gallagher et al., 2019; Huggett et al., 2016; Iwai et al., 2019; Koltun et al., 2012; Kurasawa et al., 2020; Sawa and Masai, 2009; Swords et al., 2010; Vanotti et al., 2008). The efforts to develop small-molecule inhibitors will greatly benefit from high-resolution structural information.

CDC7 possesses the canonical bilobal kinase fold, which is interrupted by unique kinase inserts (KIs). Human CDC7 harbors

 © 2020 The Authors. Published by Elsevier Ltd.
This is an open access article under the CC BY license (http://creativecommons.org/licenses/by/4.0/).

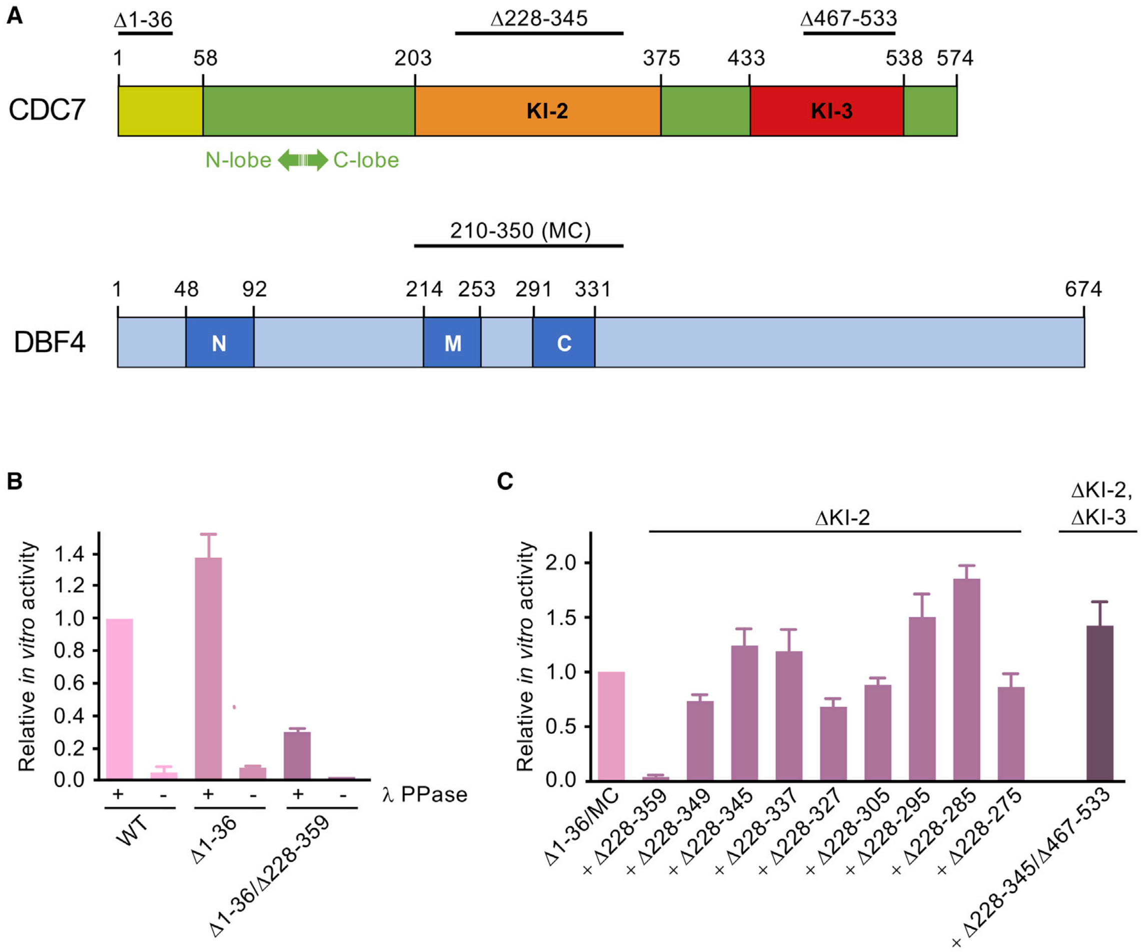

Figure 1. Design and Activity of CDC7 Deletion Constructs

(A) Schematic of human CDC7 and DBF4. Locations of kinase inserts (KI-2 and KI-3) in CDC7 and conserved motifs in DBF4 (N, M, and C) are indicated. Deletions in CDC7 (Δ1–36, Δ228–345, and Δ467–533) and the span of the DBF4 fragment used for crystallography in this work are shown above the schematics.

(B) *In vitro* kinase activities of WT full-length or deleted CDC7 constructs produced in complex with $DBF4^{MC}$ with or without treatment with λ phage protein phosphatase (λPPase). Activities were normalized to WT kinase pre-treated with phosphatase. The bar plots show mean values with standard deviations from triplicate measurements.

(C) Effects of deletions in KI-2 and KI-3 on *in vitro* kinase activities of CDC7(Δ1–36)-$DBF4^{MC}$. The KI-2 deletion used for crystallography previously (Hughes et al., 2012) is Δ228–359; the rightmost bar reports relative activity of the construct used for crystallography in the current work. All constructs were pre-treated with λPPase, and activities were normalized to the construct with full-length inserts.

Error bars represent standard deviations based on measurements done in triplicate.

two such inserts: KI-2 (residues 202–373), extending the kinase activation loop, and KI-3 (residues 433–539) separating the αG and αH helices of the C lobe (Figure 1A). DBF4 is predicted to be largely unstructured, harboring only three short conserved motifs N, M, and C (Figure 1A) (Masai and Arai, 2000). DBF4 motif N features an extended BRCT fold and is implicated in the interactions with the checkpoint kinase RAD53 and the origin recognition complex (Matthews et al., 2012). Motifs M and C, collectively comprising residues 215–330 of human DBF4, are necessary and sufficient to stimulate CDC7 kinase (Kitamura et al., 2011; Ogino et al., 2001).

A crystal structure of a heterodimeric human CDC7-DBF4 construct possessing partial kinase activity shed light on the catalytic core of the S-phase kinase (Hughes et al., 2012). The structure revealed an extensive bipartite kinase-activator interface, with DBF4 motifs C and M engaging CDC7 N and C lobes, respectively. In particular, the interactions involving DBF4 motif C are critical for orienting the canonical αC helix of CDC7. Crystallization required substantial deletions in KI-2 and KI-3 due to the inherent flexibility of these regions, and the deletion within KI-2 led to a considerable reduction of the kinase activity (Hughes et al., 2012). To identify the components of KI-2 important for the kinase activity, we determined crystal structures of a heterodimeric CDC7-DBF4 construct with improved activity in the absence and presence of a bound MCM2-derived substrate peptide. The structures reveal a Zn-binding domain in KI-2 of CDC7 that pins back the activation loop and opens up the active site for substrate binding. We also show that invariant CDC7 residues Arg373 and Arg380 are critical for the interactions with an acidic P+1 residue of the substrate.

RESULTS

Identification and Crystallization of a CDC7-DBF4 Construct with Improved Activity

The presence of extended disordered regions in CDC7 and DBF4 presents a challenge to crystallization of the heterodimer. The previously characterized construct comprised CDC7 mutant lacking the first 36 amino acids (Δ1–36), which are predicted to be disordered, in addition to the deletions of residues 228–359 in KI-2 and 484–529 in KI-3, in complex with a fragment of DBF4 spanning motifs M and C (referred to as DBF4^{MC}, residues 210–350) (Hughes et al., 2012). The heterodimer retained partial functionality in an *in vitro* kinase assay, and the loss of activity was attributed to the deletion in KI-2 (Hughes et al., 2010). To identify constructs with improved kinase function, we prepared a set of CDC7(Δ1–36)-DBF4^{MC} constructs harboring nested deletions within KI-2. Active CDC7-DBF4 constructs can undergo extensive autophosphorylation during expression in bacteria (Hughes et al., 2010), which strongly inhibits activity of the enzyme (Figure 1B). Therefore, CDC7-DBF4 heterodimers were isolated using a modified protocol, which incorporated a phosphatase treatment to reverse autophosphorylation. The proteins were tested in an *in vitro* kinase assay using a biotinylated peptide spanning residues 35–47 of human MCM2 that contains the CDC7 target residue Ser40 and is primed by phosphorylation on Ser41 (Montagnoli et al., 2006). Deletion of CDC7 KI-2 residues 228–359 resulted in over 50-fold reduction in activity (Figure 1C), confirming the importance of KI-2. The defect associated with the deletion was considerably more pronounced than reported previously with non-dephosphorylated proteins. This is not surprising, because the most catalytically robust constructs, such as the heterodimers containing complete KI-2, are subject to more efficient inhibitory auto-phosphorylation. Strikingly, adding back as few as 10 amino acid residues at the end of the KI-2 was sufficient to restore the *in vitro* kinase activity (Figure 1C).

To facilitate crystallization of the catalytically competent constructs, we replaced 22 KI-3 residues retained in the previously characterized construct, but not visible in the structure, with a pentapeptide linker Gly-Ala-Gly-Ala-Gly (the deletion designated Δ467–533). The modification did not affect the kinase activity *in vitro* (Figure 1C). Crystals were obtained with CDC7(Δ1–36/Δ228–345/Δ467–533)-DBF4^{MC} heterodimer, which retained full activity compared with the matched control harboring complete KI-2 and KI-3 (Figure 1C). X-ray diffraction data were collected using crystals grown in the presence of the ATP-competitive CDC7 inhibitor XL413 (Koltun et al., 2012). To visualize the CDC7 active site in a functional state, we used the drug-bound form to nucleate crystals in the presence of the ground-state ATP mimic ADP-BeF_3^- (Kagawa et al., 2004).

A Zinc-Binding Domain in CDC7 KI-2 Is Critical for Kinase Activity

Crystal structures of the XL413-bound and nucleotide-bound complexes were refined to 1.4 and 1.8 Å resolution, respectively (Figures 2A and S1; Table 1). The structures are highly similar to that of the previously characterized construct (Hughes et al., 2012), with the root-mean-square deviations (RMSDs) of common αC atom positions between the two constructs of ~0.6 Å (Figure S1). As reported previously, DBF4 motif C (residues 292–347) comprises a compact Zn-binding domain followed by a short α helix and an extended peptide that together wrap around the CDC7 N lobe. These interactions were previously shown to modulate the kinase activity via CDC7 αC (Hughes et al., 2012). DBF4 motif M (residues 210–254) forms a three-stranded β sheet with KI-3 and uses extended coiled regions to wrap around the C lobe of CDC7. As in the previous crystal structures, the linker region connecting DBF4 motifs C and M, comprising 37 residues, is disordered (Figure 2A).

The novel feature revealed by the crystal structures is found in the region spanning the end of KI-2 and the beginning of the CDC7 activation segment. Here, residues Cys351, Cys353, Cys360, and Cys363, all which are invariant among metazoan CDC7 orthologs, coordinate a single metal ion, nucleating zinc-finger (ZF) domain. We confirmed the presence of a Zn atom in the structure by anomalous diffraction (Figure 2B). The ZF domain nestles on motif M of DBF4, sandwiching it against the C lobe of CDC7 (Figures 2A and 2B). Crucially, this interaction pins back the beginning of the CDC7 activation loop, inducing it to adopt a stable conformation. Disruption of the ZF domain by a more extensive deletion that removed two of the four Zn^{2+} coordinating Cys residues explains why the activation loop was only partially ordered in the previously reported structure. In agreement with the importance of the ZF domain, substitutions of any of the four metal coordinating residues in the context of the complete KI-2 substantially reduced the kinase activity *in vitro* (Figure 2B).

Structural Basis for the Selectivity of CDC7 for Substrates with Acidic Residues at P+1 Position

The organization of the CDC7 active site in complex with ADP-BeF_3^- is strikingly similar to that observed in the substrate transition complex of CDK2 (Bao et al., 2011). A pair of octahedrally coordinated Mg^{2+} ions is observed in contact with the BeF_3^- moiety that mimics the γ-phosphate of the nucleotide substrate (Figures 2C and S2A). Given that both the catalytic center and the activation loop, which underlies the substrate-binding platform, are well ordered in the structure, we carried out extensive screening to obtain crystals of the kinase bound to a target peptide. Co-crystallization was only successful using the bisubstrate approach (Parang and Cole, 2002) with MCM2(33–47) peptide covalently conjugated to ATPγS via bromoacetyl-aminoalanine in place of Ser40 and pre-phosphorylated on Ser41. X-ray diffraction data were collected to 1.7-Å resolution (Table 1), and the structure was solved by molecular replacement. The nucleotide bound in the active site and nine residues of the substrate peptide, spanning MCM2 residues 38–46, were built in the resulting electron density map (Figures 3A and S2B). No density could be attributed to the γ-phosphate moiety, presumably due to hydrolysis of the bisubstrate ligand during crystallization. Therefore, the present ligands were interpreted as ADP and the thioacetyl adduct of the MCM2 peptide (Figures 3A and S2B).

The CDC7 target site consensus is limited to an acidic or pre-phosphorylated residue at substrate P+1 position (Charych et al., 2008; Cho et al., 2006). In the crystal structure, the

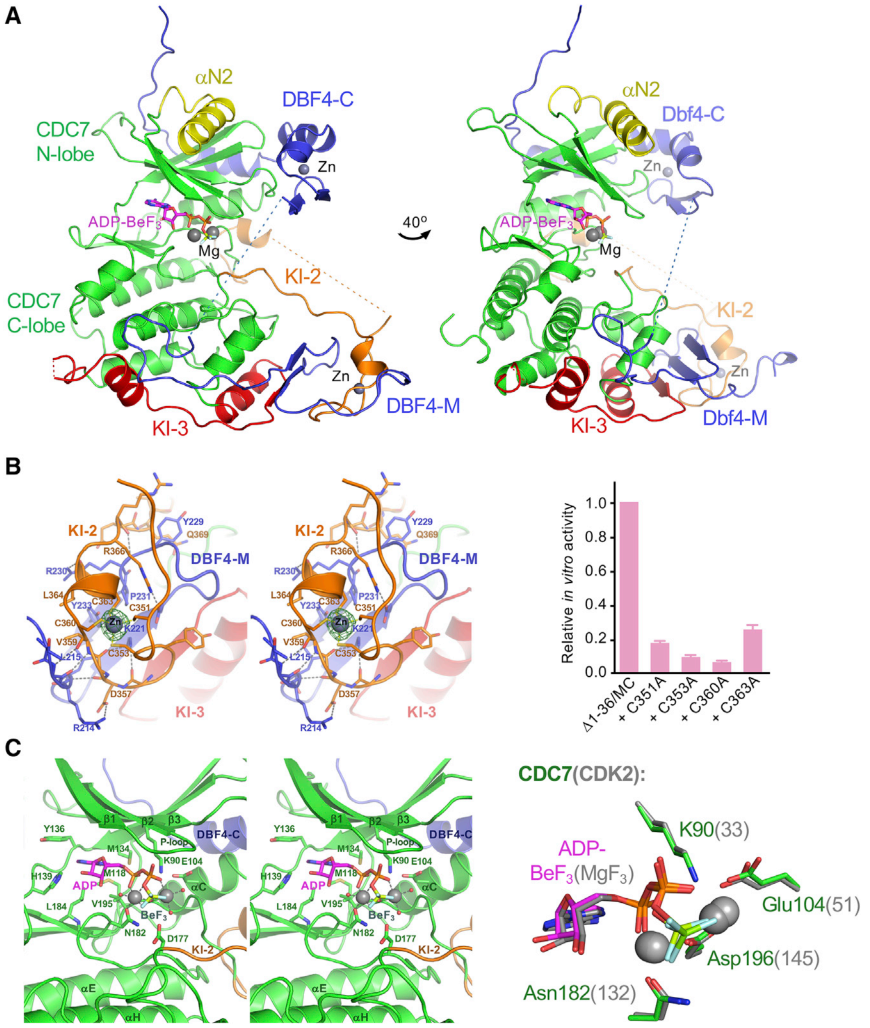

Figure 2. Crystal Structure of CDC7(Δ1–36/Δ228–345/Δ467–533)-DBF4^{MC} Heterodimer in Complex with ADP-BeF$_3^-$

(A) Overview of the structure. Protein chains are shown as cartoons and colored as in Figure 1A, with DBF4 in blue, canonical kinase CDC7 regions in green, and unique elements in yellow (N-terminal region), orange (KI-2), or red (KI-3). Zn and Mg atoms are shown as gray spheres, and nucleotide (ADP-BeF$_3$) as sticks, with carbon atoms in pink and the remaining atoms according to standard conventions: nitrogen, oxygen, phosphorus, beryllium, and fluoride atoms in blue, red, orange, light green, and gray, respectively. Dotted lines show connectivity, where peptide linkages are disordered in the crystal structure. See Figure S1 for comparison with the structure of the previously reported construct (Hughes et al., 2012).

(B) Left: stereo view of the KI-2 ZF domain and its interactions with DBF4. Selected residues are shown as sticks and indicated. Anomalous difference map calculated from diffraction data acquired using X-rays at the absorption K-edge of Zn (λ = 1.2837 Å) is shown as green mesh, contoured at 5σ; the peak height around the Zn atom is >30σ. Right: relative kinase activities of CDC7(Δ1–36)-DBF4^{MC} constructs without (leftmost bar) and with Ala substitutions of the Zn-coordinating Cys residues.

(C) Left: details of the active site. Final 2F$_o$-F$_c$ electron density map for the active-site region is shown in Figure S2A. Right: superposition of the active sites of CDC7 and CDK2 (PDB: 3QHW) active sites bound to ATP analogs. Active-site Mg atoms are shown as gray spheres. Nucleotide analogs and selected amino acid residues are shown as sticks and labeled, colored as in (A) (CDC7) or with carbon atoms in gray (CDK2).

phosphate group on Ser41 of the target peptide, mimicking priming by CDK2, forms well-defined interactions with side chains of Arg373 and Arg380 (Figure 3A), invariant residues from the CDC7 activation loop and the C lobe, respectively. Accordingly, substitution of either or both of these residues to Ala compromised the kinase activity (Figure 3B). In addition to the interactions involving MCM2 phospho-Ser41, the side chain of Arg44, located in P+4 substrate position, is buried in a pocket lined by CDC7 and DBF4 residues. Within the pocket, MCM2 Arg44 makes a salt bridge with DBF4 Glu297 and hydrogen bonds with Ser96 and Gly198 of CDC7, stacking against CDC7 Phe96 (Figure 3A). However, the CDC7 target site consensus does not show conservation at P+4 position (Charych et al., 2008; Cho et al., 2006), suggesting that the pocket may not be very selective. Concordantly, substitutions of Arg44 in the context of the substrate peptide resulted in relatively minor defects of the *in vitro* kinase activity, in sharp contrast to the near complete loss of phosphorylation upon removal of the priming phosphate on Ser41 (Figure 3C). These results further confirm the importance of the P+1 position as the major determinant for substrate specificity around the CDC7 active site.

DISCUSSION

DBF4 motifs M and C are collectively required to support CDC7 activity (Kitamura et al., 2011; Ogino et al., 2001), and we reported previously that binding of DBF4 motif C to the N lobe of CDC7 stabilizes the functional ‘‘in’’ conformation of αC (Hughes et al., 2012), which is a recurrent mechanism of protein kinase regulation (Bayliss et al., 2003; Jeffrey et al., 1995; Sessa et al., 2005; Yang et al., 2002). The crystal structures described here reveal the unique role of DBF4 motif M: through the interaction with motif M, the KI-2 ZF domain pins the beginning of the CDC7 activation loop to the kinase C lobe, allowing it to adopt a stable conformation (Figures 2A, 2B, and S1). Accordingly, substitutions of the Zn-coordinating Cys residues in the ZF domain affected the kinase activity *in vitro* (Figure 2B). A disruption of this domain was likely responsible for the reduced activity of the previously characterized construct (Hughes et al., 2012). The interactions involving the KI-2 ZF domain appear to functionally substitute those formed by phosphorylated residues in the kinases that are activated by phosphorylation of their activation segments.

Table 1. X-Ray Data Collection and Refinement Statistics

Ligand	XL-413	ADP-BeF_3^-	ATPγS-S40-MCM2(33–47)
PDB accession code	6YA6	6YA8	6YA7
Data Collection[a]			
Wavelength (Å)	1.0332	0.97957	0.97625
Space group	$P4_12_12$	$P4_12_12$	$P4_12_12$
Unit cell parameters			
a, *b*, *c* (Å)	61.64, 61.64, 233.91	61.15, 61.15, 235.44	62.28, 62.28, 234.79
α, β, γ (°)	90, 90, 90	90, 90, 90	90, 90, 90
No. of crystals used	1	1	1
Resolution (Å)	59.61–1.44 (1.491–1.44)	58.86–1.79 (1.84–1.79)	60.2–1.67 (1.71–1.67)
No. of reflections			
Measured	750,955	493,033	665,579
Unique	82,613	43,037	54,941
Completeness (%)	99.6 (95.7)	99.1 (90.2)	99.96 (99.8)
Multiplicity	9.1 (6.4)	11.5 (7.2)	12.1 (8.5)
$<I/\sigma(I)>$	26.4 (2.7)	23.6 (2.8)	19.4 (3.3)
R_{merge}	0.049 (0.584)	0.067 (0.614)	0.098 (0.724)
$CC_{1/2}$	1.000 (0.77)	0.999 (0.817)	0.999 (0.81)
Refinement Statistics			
Resolution range (Å)	42.85–1.44	48.24–1.79	58.7–1.67
No. of reflections			
Total	82,484	42,929	54,918
Free	4,139	2,208	2,739
R_{work}/R_{free}	0.1666/ 0.1933	0.1619/ 0.1937	0.1562/ 0.1852
No. of atoms:			
Total	4,072	3,917	4,135
Protein	3,569	3,580	3,733
Ligands	39	35	36
Solvent	464	302	366
RMSDs from ideal			
Bond lengths (Å)	0.006	0.007	0.010
Bond angles (°)	0.87	0.88	1.15
Average *B* factor ($Å^2$)	20.95	26.57	25.08
Clashscore[b]	1.66	2.49	3.99
Favored rotamers[b]	98.22	97.22	97.08
Ramachandran plot (%)[b]			
Favored	97.70	98.14	97.79
Disallowed	0.23	0	0

[a]Values in parentheses correspond to the highest-resolution bin.
[b]Analyzed using MolProbity (http://molprobity.biochem.duke.edu/).

We conclude that CDC7 activity is controlled by DBF4 at two levels: (1) via ordering of the activation loop dependent on the ZF domain-DBF4 motif M interactions; and (2) via the control of αC insertion through by DBF4 motif C (Hughes et al., 2012). Of note, the flexible linkage of DBF4 motifs M and C (indeed, the 34-residue region separating them is disordered in our crystal structures) may allow additional finesse in the control of CDC7 activity. Sandwiched between CDC7 C lobe and KI-2 ZF domain, DBF4 motif M appears stably tethered to CDC7, possibly allowing motif C to engage and disengage from the N lobe without dissociation of the holoenzyme. Intriguingly, the Cys tetrad involved in Zn coordination is invariant across metazoan CDC7 orthologs but not in budding yeast. It is conceivable that a different mechanism is used for pinning back CDC7 activation loop by yeast DBF4. An interesting observation of this study was the apparent lack of requirement for significant portions of KI2 and KI3 for CDC7 kinase activity *in vitro*. The inserts, universally present in CDC7 orthologs, may participate in engaging binding partners and/or regulating various cellular functions of the kinase. Indeed, roles for KI-2 and KI-3 in nuclear import and export of CDC7 have been proposed (Kim et al., 2007; Kim and Lee, 2006).

The ordering of the activation loop allowed co-crystallization of the kinase in complex with a target peptide. In our hands, a bisubstrate construct containing an MCM2-derived peptide covalently linked to ATPγS was invaluable in obtaining the crystals. Similar approaches facilitated co-crystallization of other protein kinases with their substrates and are being explored in kinase inhibitor development (Cozza et al., 2015; Lavogina et al., 2010; Parang and Cole, 2002; Parang et al., 2001; Ricouart et al., 1991). Although in the crystal structure the chimeric molecule was found in a hydrolyzed state, screening using unlinked peptide derivatives have not produced co-crystals. The substrate-bound structure revealed that CDC7 Arg373 and Arg380 make favorable interactions with substrate phosphorylated Ser41. These residues are invariant across CDC7 orthologs, suggesting a conserved mechanism for the recognition of the P+1 substrate residue, which is essential for the CDC7 kinase activity (Figure 3B). Additionally, the co-crystal structure revealed interactions involving MCM2 Arg44, a residue in the P+4 substrate position (Figure 3A). This observation is intriguing given that CDK2, which was implicated in priming at least some CDC7 target sites (Montagnoli et al., 2006), has a notable preference for Lys or Arg at its substrate P+3 position (Songyang et al., 1994). Thus, it is tempting to speculate that CDC7 evolved to work in tandem during activation of replication origins by the cyclin-dependent kinase(s). However, while the pocket is suited for accepting an Arg side chain, CDC7 does not appear to discriminate against target peptides with alternative residues in P+4 position (Figure 3C). Indeed, the interactions between the kinase and the P+1 residue had greater effects on the kinase activity (Figures 3B and 3C), further highlighting the importance of P+1 position within the limited target consensus sequence of CDC7. Our results reveal that both kinase inserts present in human CDC7, KI-2 and KI-3, mediate the interaction with DBF4 motif M (Figure 2A). However, extensive portions of both inserts are either missing or

CellPress
OPEN ACCESS

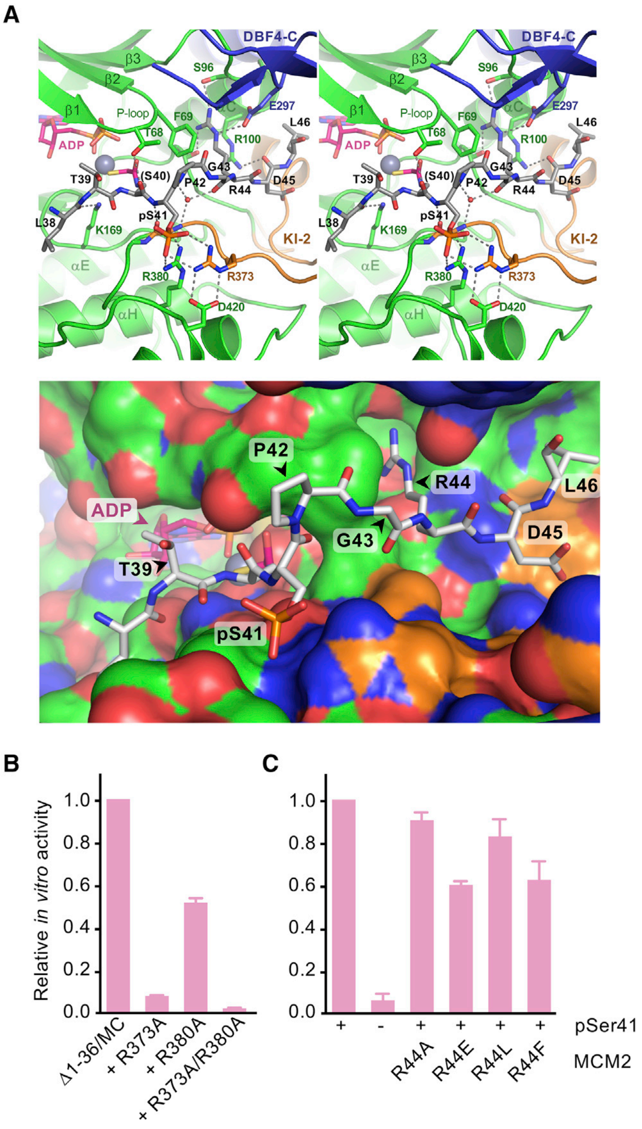

Figure 3. Structural Basis for Substrate Binding by CDC7

(A) Top: details of the interactions between ATPγS-S40-MCM2(33–47) bisubstrate adduct and CDC7. Note that the γ-phosphate moiety of the input bisubstrate has been hydrolyzed; see Figure S2B for the final $2F_o$-F_c electron density map for that region of the structure. The substrate peptide is shown as sticks with carbon atoms in gray. Dotted lines indicate hydrogen-bond interactions. Bottom: same region of the structure, with CDC7 and DBF4 in surface representation.

(B) Relative kinase activities of CDC7(Δ1–36)-DBF4^{MC} constructs without (leftmost bar) or with Ala substitutions of Arg373 and/or Arg380.

(C) Relative activities of CDC7(Δ1–36)-DBF4^{MC} on MCM2(35–47) peptides with (all except second bar from the left) or without priming phosphorylation on Ser41 (second bar). Amino acid substitutions of Arg44 in substrate peptides are indicated below the plot.

disordered in the crystallized constructs, and further work will be required to ascertain their roles. The current structures will be informative for the design of competitive active-site antagonists of CDC7 kinase. Furthermore, the multivalent CDC7-DBF4 interface described here may be utilized for the development of highly targeted non-competitive inhibitors.

STAR★METHODS

Detailed methods are provided in the online version of this paper and include the following:

- KEY RESOURCES TABLE
- RESOURCE AVAILABILITY
  - Lead Contact
  - Materials Availability
  - Data and Code Availability
- EXPERIMENTAL MODEL AND SUBJECT DETAILS
- METHOD DETAILS
  - DNA Constructs
  - Recombinant Proteins
  - Peptides and Bisubstrates
  - *In Vitro* Kinase Activity Assays
  - Crystallisation, X-Ray Data Collection and Structure Refinement
- QUANTIFICATION AND STATISTICAL ANALYSIS

SUPPLEMENTAL INFORMATION

Supplemental Information can be found online at https://doi.org/10.1016/j.str.2020.05.010.

ACKNOWLEDGMENTS

We thank P. Walker and A. Purkiss for help with protein crystallography and software support, and Dr. Hassan Belrhali and the staff of BM14 (European Synchrotron Light Source, Grenoble, France) and I03 (Diamond Light Source, Oxfordshire, UK) for assistance with data collection. This work was funded jointly by Cancer Research UK, the Wellcome Trust, and the UK Medical Research Council at the Francis Crick Institute (FC001061).

AUTHOR CONTRIBUTIONS

Experimental design: P.C.; DNA cloning, protein expression, purification, and biochemistry: S.D.D. and S.M.H.; Peptide and bisubstrate synthesis: S.F. and N.O'R.; Crystallography: S.D.D.; X-ray data collection and processing, model building, and refinement: S.D.D., P.C., and V.E.P.; Manuscript preparation: P.C. and S.D.D., with input from all authors.

DECLARATION OF INTERESTS

The authors declare no competing interests.

Received: March 18, 2020
Revised: May 11, 2020
Accepted: May 19, 2020
Published: June 9, 2020

REFERENCES

Adams, P.D., Afonine, P.V., Bunkoczi, G., Chen, V.B., Davis, I.W., Echols, N., Headd, J.J., Hung, L.W., Kapral, G.J., Grosse-Kunstleve, R.W., et al. (2010). PHENIX: a comprehensive Python-based system for macromolecular structure solution. Acta Crystallogr. D Biol. Crystallogr. *66*, 213–221.

Bao, Z.Q., Jacobsen, D.M., and Young, M.A. (2011). Briefly bound to activate: transient binding of a second catalytic magnesium activates the structure and dynamics of CDK2 kinase for catalysis. Structure *19*, 675–690.

Bayliss, R., Sardon, T., Vernos, I., and Conti, E. (2003). Structural basis of Aurora-A activation by TPX2 at the mitotic spindle. Mol. Cell *12*, 851–862.

Bonte, D., Lindvall, C., Liu, H., Dykema, K., Furge, K., and Weinreich, M. (2008). Cdc7-Dbf4 kinase overexpression in multiple cancers and tumor cell lines is correlated with p53 inactivation. Neoplasia *10*, 920–931.

Brandao, L.N., Ferguson, R., Santoro, I., Jinks-Robertson, S., and Sclafani, R.A. (2014). The role of Dbf4-dependent protein kinase in DNA polymerase zeta-dependent mutagenesis in Saccharomyces cerevisiae. Genetics *197*, 1111–1122.

Buck, V., White, A., and Rosamond, J. (1991). Cdc7 protein-kinase activity is required for mitosis and meiosis in *Saccharomyces cerevisiae*. Mol. Gen. Genet. *227*, 452–457.

Cao, J.X., and Lu, Y. (2019). Targeting CDC7 improves sensitivity to chemotherapy of esophageal squamous cell carcinoma. Onco Targets Ther. *12*, 63–74.

Charych, D.H., Coyne, M., Yabannavar, A., Narberes, J., Chow, S., Wallroth, M., Shafer, C., and Walter, A.O. (2008). Inhibition of Cdc7/Dbf4 kinase activity affects specific phosphorylation sites on MCM2 in cancer cells. J. Cell. Biochem. *104*, 1075–1086.

Cheng, A.N., Jiang, S.S., Fan, C.C., Lo, Y.K., Kuo, C.Y., Chen, C.H., Liu, Y.L., Lee, C.C., Chen, W.S., Huang, T.S., et al. (2013). Increased Cdc7 expression is a marker of oral squamous cell carcinoma and overexpression of Cdc7 contributes to the resistance to DNA-damaging agents. Cancer Lett. *337*, 218–225.

Cheng, A.N., Lo, Y.K., Lin, Y.S., Tang, T.K., Hsu, C.H., Hsu, J.T., and Lee, A.Y. (2018). Identification of novel Cdc7 kinase inhibitors as anti-cancer agents that target the interaction with Dbf4 by the fragment complementation and drug repositioning approach. EBioMedicine *36*, 241–251.

Cho, W.H., Lee, Y.J., Kong, S.I., Hurwitz, J., and Lee, J.K. (2006). CDC7 kinase phosphorylates serine residues adjacent to acidic amino acids in the minichromosome maintenance 2 protein. Proc. Natl. Acad. Sci. U S A *103*, 11521–11526.

Clarke, L.E., Fountaine, T.J., Hennessy, J., Bruggeman, R.D., Clarke, J.T., Mauger, D.T., and Helm, K.F. (2009). Cdc7 expression in melanomas, Spitz tumors and melanocytic nevi. J. Cutan. Pathol. *36*, 433–438.

Cordingley, M.G., Callahan, P.L., Sardana, V.V., Garsky, V.M., and Colonno, R.J. (1990). Substrate requirements of human rhinovirus 3C protease for peptide cleavage in vitro. J. Biol. Chem. *265*, 9062–9065.

Costanzo, V., Shechter, D., Lupardus, P.J., Cimprich, K.A., Gottesman, M., and Gautier, J. (2003). An ATR- and Cdc7-dependent DNA damage checkpoint that inhibits initiation of DNA replication. Mol. Cell *11*, 203–213.

Cozza, G., Zanin, S., Sarno, S., Costa, E., Girardi, C., Ribaudo, G., Salvi, M., Zagotto, G., Ruzzene, M., and Pinna, L.A. (2015). Design, validation and efficacy of bisubstrate inhibitors specifically affecting ecto-CK2 kinase activity. Biochem. J. *471*, 415–430.

Duncker, B.P., Shimada, K., Tsai-Pflugfelder, M., Pasero, P., and Gasser, S.M. (2002). An N-terminal domain of Dbf4p mediates interaction with both origin recognition complex (ORC) and Rad53p and can deregulate late origin firing. Proc. Natl. Acad. Sci. U S A *99*, 16087–16092.

Emsley, P., and Cowtan, K. (2004). Coot: model-building tools for molecular graphics. Acta Crystallogr. D Biol. Crystallogr. *60*, 2126–2132.

Evans, P. (2006). Scaling and assessment of data quality. Acta Crystallogr. D Biol. Crystallogr. *62*, 72–82.

Ferreira, M.G., Santocanale, C., Drury, L.S., and Diffley, J.F.X. (2000). Dbf4p, an essential S phase-promoting factor, is targeted for degradation by the anaphase-promoting complex. Mol. Cell. Biol. *20*, 242–248.

Gad, S.A., Ali, H.E.A., Gaballa, R., Abdelsalam, R.M., Zerfaoui, M., Ali, H.I., Salama, S.H., Kenawy, S.A., Kandil, E., and Abd Elmageed, Z.Y. (2019). Targeting CDC7 sensitizes resistance melanoma cells to BRAF(V600E)-specific inhibitor by blocking the CDC7/MCM2-7 pathway. Sci. Rep. *9*, 14197.

Gallagher, P.F., Coyle, V., Evans, T.R.J., Plummer, E.R., Clive, S., McGuigan, L., Roxburgh, P., Haris, N.M., Symeonides, S.N., Naylor, G., et al. (2019). A CRUK first-in-human phase I trial of a CDC7 Inhibitor, LY3143921 hydrate, in patients with advanced solid tumors. J. Clin. Oncol. *37*, TPS3167.

Ghatalia, P., Yang, E.S., Lasseigne, B.N., Ramaker, R.C., Cooper, S.J., Chen, D., Sudarshan, S., Wei, S., Guru, A.S., Zhao, A., et al. (2016). Kinase gene expression profiling of metastatic clear cell renal cell carcinoma tissue identifies potential new therapeutic targets. PLoS One *11*, e0160924.

Hou, Y., Wang, H.Q., and Ba, Y. (2012). High expression of cell division cycle 7 protein correlates with poor prognosis in patients with diffuse large B-cell lymphoma. Med. Oncol. *29*, 3498–3503.

Huggett, M.T., Tudzarova, S., Proctor, I., Loddo, M., Keane, M.G., Stoeber, K., Williams, G.H., and Pereira, S.P. (2016). Cdc7 is a potent anti-cancer target in pancreatic cancer due to abrogation of the DNA origin activation checkpoint. Oncotarget *7*, 18495–18507.

Hughes, S., Elustondo, F., Di Fonzo, A., Leroux, F.G., Wong, A.C., Snijders, A.P., Matthews, S.J., and Cherepanov, P. (2012). Crystal structure of human CDC7 kinase in complex with its activator DBF4. Nat. Struct. Mol. Biol. *19*, 1101–1107.

Hughes, S., Jenkins, V., Dar, M.J., Engelman, A., and Cherepanov, P. (2010). Transcriptional co-activator LEDGF interacts with Cdc7-activator of S-phase kinase (ASK) and stimulates its enzymatic activity. J. Biol. Chem. *285*, 541–554.

Im, J.S., and Lee, J.K. (2008). ATR-dependent activation of p38 MAP kinase is responsible for apoptotic cell death in cells depleted of Cdc7. J. Biol. Chem. *283*, 25171–25177.

Ito, S., Ishii, A., Kakusho, N., Taniyama, C., Yamazaki, S., Fukatsu, R., Sakaue-Sawano, A., Miyawaki, A., and Masai, H. (2012). Mechanism of cancer cell death induced by depletion of an essential replication regulator. PLoS One *7*, e36372.

Iwai, K., Nambu, T., Dairiki, R., Ohori, M., Yu, J., Burke, K., Gotou, M., Yamamoto, Y., Ebara, S., Shibata, S., et al. (2019). Molecular mechanism and potential target indication of TAK-931, a novel CDC7-selective inhibitor. Sci. Adv. *5*, eaav3660.

Jaafari-Ashkavandi, Z., Ashraf, M.J., and Abbaspoorfard, A.A. (2019). Overexpression of CDC7 in malignant salivary gland tumors correlates with tumor differentiation. Braz. J. Otorhinolaryngol. *85*, 144–149.

Jackson, A.L., Pahl, P.M.B., Harrison, K., Rosamond, J., and Sclafani, R.A. (1993). Cell-cycle regulation of the yeast Cdc7 protein-kinase by association with the Dbf4 protein. Mol. Cell. Biol. *13*, 2899–2908.

Jeffrey, P.D., Russo, A.A., Polyak, K., Gibbs, E., Hurwitz, J., Massague, J., and Pavletich, N.P. (1995). Mechanism of CDK activation revealed by the structure of a cyclinA-CDK2 complex. Nature *376*, 313–320.

Kabsch, W. (2010). XDS. Acta Crystallogr. D Biol. Crystallogr. *66*, 125–132.

Kagawa, R., Montgomery, M.G., Braig, K., Leslie, A.G., and Walker, J.E. (2004). The structure of bovine F1-ATPase inhibited by ADP and beryllium fluoride. EMBO J. *23*, 2734–2744.

Katis, V.L., Lipp, J.J., Imre, R., Bogdanova, A., Okaz, E., Habermann, B., Mechtler, K., Nasmyth, K., and Zachariae, W. (2010). Rec8 phosphorylation by casein kinase 1 and Cdc7-Dbf4 kinase regulates cohesin cleavage by separase during meiosis. Dev. Cell *18*, 397–409.

Kim, B.J., Kim, S.Y., and Lee, H. (2007). Identification and characterization of human Cdc7 nuclear retention and export sequences in the context of chromatin binding. J. Biol. Chem. *282*, 30029–30038.

Kim, B.J., and Lee, H. (2006). Importin-beta mediates Cdc7 nuclear import by binding to the kinase insert II domain, which can be antagonized by importin-alpha. J. Biol. Chem. *281*, 12041–12049.

Kitamura, R., Fukatsu, R., Kakusho, N., Cho, Y.S., Taniyama, C., Yamazaki, S., Toh, G.T., Yanagi, K., Arai, N., Chang, H.J., et al. (2011). Molecular mechanism of activation of human Cdc7 kinase: bipartite interaction with Dbf4/activator of S phase kinase (ASK) activation subunit stimulates ATP binding and substrate recognition. J. Biol. Chem. *286*, 23031–23043.

Koltun, E.S., Tsuhako, A.L., Brown, D.S., Aay, N., Arcalas, A., Chan, V., Du, H., Engst, S., Ferguson, K., Franzini, M., et al. (2012). Discovery of XL413, a potent and selective CDC7 inhibitor. Bioorg. Med. Chem. Lett. *22*, 3727–3731.

Kulkarni, A.A., Kingsbury, S.R., Tudzarova, S., Hong, H.K., Loddo, M., Rashid, M., Rodriguez-Acebes, S., Prevost, A.T., Ledermann, J.A., Stoeber, K., et al. (2009). Cdc7 kinase is a predictor of survival and a novel therapeutic target in epithelial ovarian carcinoma. Clin. Cancer Res. *15*, 2417–2425.

Kurasawa, O., Miyazaki, T., Homma, M., Oguro, Y., Imada, T., Uchiyama, N., Iwai, K., Yamamoto, Y., Ohori, M., Hara, H., et al. (2020). Discovery of a novel, highly potent, and selective thieno[3,2-d]pyrimidinone-based Cdc7 inhibitor

with a quinuclidine moiety (TAK-931) as an orally active investigational antitumor agent. J. Med. Chem. *63*, 1084–1104.

Lavogina, D., Enkvist, E., and Uri, A. (2010). Bisubstrate inhibitors of protein kinases: from principle to practical applications. ChemMedChem *5*, 23–34.

Lo, H.C., Kunz, R.C., Chen, X., Marullo, A., Gygi, S.P., and Hollingsworth, N.M. (2012). Cdc7-Dbf4 is a gene-specific regulator of meiotic transcription in yeast. Mol. Cell. Biol *32*, 541–557.

Masai, H., and Arai, K. (2000). Dbf4 motifs: conserved motifs in activation subunits for Cdc7 kinases essential for S-phase. Biochem. Biophys. Res. Commun. *275*, 228–232.

Masai, H., Taniyama, C., Ogino, K., Matsui, E., Kakusho, N., Matsumoto, S., Kim, J.M., Ishii, A., Tanaka, T., Kobayashi, T., et al. (2006). Phosphorylation of MCM4 by Cdc7 kinase facilitates its interaction with Cdc45 on the chromatin. J. Biol. Chem. *281*, 39249–39261.

Matos, J., Lipp, J.J., Bogdanova, A., Guillot, S., Okaz, E., Junqueira, M., Shevchenko, A., and Zachariae, W.G. (2008). Dbf4-Dependent Cdc7 kinase links DNA replication to the segregation of homologous chromosomes in meiosis I. Cell *135*, 662–678.

Matthews, L.A., Jones, D.R., Prasad, A.A., Duncker, B.P., and Guarne, A. (2012). Saccharomyces cerevisiae Dbf4 has unique fold necessary for interaction with Rad53 kinase. J. Biol. Chem. *287*, 2378–2387.

McCoy, A.J., Grosse-Kunstleve, R.W., Adams, P.D., Winn, M.D., Storoni, L.C., and Read, R.J. (2007). Phaser crystallographic software. J. Appl. Crystallogr. *40*, 658–674.

Melling, N., Muth, J., Simon, R., Bokemeyer, C., Terracciano, L., Sauter, G., Izbicki, J.R., and Marx, A.H. (2015). Cdc7 overexpression is an independent prognostic marker and a potential therapeutic target in colorectal cancer. Diagn. Pathol. *10*, 125.

Miller, C.T., Gabrielse, C., Chen, Y.C., and Weinreich, M. (2009). Cdc7p-Dbf4p regulates mitotic exit by inhibiting Polo kinase. PLoS Genet. *5*, e1000498.

Mishra, P.K., Ciftci-Yilmaz, S., Reynolds, D., Au, W.C., Boeckmann, L., Dittman, L.E., Jowhar, Z., Pachpor, T., Yeh, E., Baker, R.E., et al. (2016). Polo kinase Cdc5 associates with centromeres to facilitate the removal of centromeric cohesin during mitosis. Mol. Biol. Cell *27*, 2286–2300.

Montagnoli, A., Tenca, P., Sola, F., Carpani, D., Brotherton, D., Albanese, C., and Santocanale, C. (2004). Cdc7 inhibition reveals a p53-dependent replication checkpoint that is defective in cancer cells. Cancer Res. *64*, 7110–7116.

Montagnoli, A., Valsasina, B., Brotherton, D., Troiani, S., Rainoldi, S., Tenca, P., Molinari, A., and Santocanale, C. (2006). Identification of Mcm2 phosphorylation sites by S-phase-regulating kinases. J. Biol. Chem. *281*, 10281–10290.

Murakami, H., and Keeney, S. (2014). Temporospatial coordination of meiotic DNA replication and recombination via DDK recruitment to replisomes. Cell *158*, 861–873.

Murshudov, G.N., Vagin, A.A., and Dodson, E.J. (1997). Refinement of macromolecular structures by the maximum-likelihood method. Acta Crystallogr. D Biol. Crystallogr. *53*, 240–255.

Natsume, T., Muller, C.A., Katou, Y., Retkute, R., Gierlinski, M., Araki, H., Blow, J.J., Shirahige, K., Nieduszynski, C.A., and Tanaka, T.U. (2013). Kinetochores coordinate pericentromeric cohesion and early DNA replication by Cdc7-Dbf4 kinase recruitment. Mol. Cell *50*, 661–674.

O' Reilly, E., Dhami, S.P.S., Baev, D.V., Ortutay, C., Halpin-McCormick, A., Morrell, R., Santocanale, C., Samali, A., Quinn, J., O'Dwyer, M.E., et al. (2018). Repression of Mcl-1 expression by the CDC7/CDK9 inhibitor PHA-767491 overcomes bone marrow stroma-mediated drug resistance in AML. Sci. Rep. *8*, 15752.

Ogino, K., Hirota, K., Matsumoto, S., Takeda, T., Ohta, K., Arai, K., and Masai, H. (2006). Hsk1 kinase is required for induction of meiotic dsDNA breaks without involving checkpoint kinases in fission yeast. Proc. Natl. Acad. Sci. U S A *103*, 8131–8136.

Ogino, K., Takeda, T., Matsui, E., Iiyama, H., Taniyama, C., Arai, K., and Masai, H. (2001). Bipartite binding of a kinase activator activates Cdc7-related kinase essential for S phase. J. Biol. Chem. *276*, 31376–31387.

Oshiro, G., Owens, J.C., Shellman, Y., Sclafani, R.A., and Li, J.J. (1999). Cell cycle control of Cdc7p kinase activity through regulation of Dbf4p stability. Mol. Cell. Biol. *19*, 4888–4896.

Parang, K., and Cole, P.A. (2002). Designing bisubstrate analog inhibitors for protein kinases. Pharmacol. Ther. *93*, 145–157.

Parang, K., Till, J.H., Ablooglu, A.J., Kohanski, R.A., Hubbard, S.R., and Cole, P.A. (2001). Mechanism-based design of a protein kinase inhibitor. Nat. Struct. Biol. *8*, 37–41.

Princz, L.N., Wild, P., Bittmann, J., Aguado, F.J., Blanco, M.G., Matos, J., and Pfander, B. (2017). Dbf4-dependent kinase and the Rtt107 scaffold promote Mus81-Mms4 resolvase activation during mitosis. EMBO J. *36*, 664–678.

Randell, J.C.W., Fan, A., Chan, C., Francis, L.I., Heller, R.C., Galani, K., and Bell, S.P. (2010). Mec1 is one of multiple kinases that prime the Mcm2-7 helicase for phosphorylation by Cdc7. Mol. Cell *40*, 353–363.

Ricouart, A., Gesquiere, J.C., Tartar, A., and Sergheraert, C. (1991). Design of potent protein kinase inhibitors using the bisubstrate approach. J. Med. Chem. *34*, 73–78.

Rossbach, D., and Sclafani, R.A. (2016). Role of DDK in replication initiation. In The Initiation of DNA Replication in Eukaryotes, D.L. Kaplan, ed. (Springer), pp. 279–296.

Sasanuma, H., Hirota, K., Fukuda, T., Kakusho, N., Kugou, K., Kawasaki, Y., Shibata, T., Masai, H., and Ohta, K. (2008). Cdc7-dependent phosphorylation of Mer2 facilitates initiation of yeast meiotic recombination. Genes Dev. *22*, 398–410.

Sawa, M., and Masai, H. (2009). Drug design with Cdc7 kinase: a potential novel cancer therapy target. Drug Des. Devel. Ther. *2*, 255–264.

Sessa, F., Mapelli, M., Ciferri, C., Tarricone, C., Areces, L.B., Schneider, T.R., Stukenberg, P.T., and Musacchio, A. (2005). Mechanism of Aurora B activation by INCENP and inhibition by hesperadin. Mol. Cell *18*, 379–391.

Sheu, Y.J., and Stillman, B. (2006). Cdc7-Dbf4 phosphorylates MCM proteins via a docking site-mediated mechanism to promote S phase progression. Mol. Cell *24*, 101–113.

Sheu, Y.J., and Stillman, B. (2010). The Dbf4-Cdc7 kinase promotes S phase by alleviating an inhibitory activity in Mcm4. Nature *463*, 113–117.

Songyang, Z., Blechner, S., Hoagland, N., Hoekstra, M.F., Piwnica-Worms, H., and Cantley, L.C. (1994). Use of an oriented peptide library to determine the optimal substrates of protein kinases. Curr. Biol. *4*, 973–982.

Subramanian, C., and Cohen, M.S. (2019). Over expression of DNA damage and cell cycle dependent proteins are associated with poor survival in patients with adrenocortical carcinoma. Surgery *165*, 202–210.

Swords, R., Mahalingam, D., O'Dwyer, M., Santocanale, C., Kelly, K., Carew, J., and Giles, F. (2010). Cdc7 kinase—a new target for drug development. Eur. J. Cancer *46*, 33–40.

Takahashi, T.S., Basu, A., Bermudez, V., Hurwitz, J., and Walter, J.C. (2008). Cdc7-Drf1 kinase links chromosome cohesion to the initiation of DNA replication in Xenopus egg extracts. Genes Dev. *22*, 1894–1905.

Terwilliger, T.C., Grosse-Kunstleve, R.W., Afonine, P.V., Moriarty, N.W., Zwart, P.H., Hung, L.W., Read, R.J., and Adams, P.D. (2008). Iterative model building, structure refinement and density modification with the PHENIX AutoBuild wizard. Acta Crystallogr. D Biol. Crystallogr. *64*, 61–69.

Tsuji, T., Lau, E., Chiang, G.G., and Jiang, W. (2008). The role of Dbf4/Drf1-dependent kinase Cdc7 in DNA-damage checkpoint control. Mol. Cell *32*, 862–869.

Ullah, R., Shah, M.A., Tufail, S., Ismat, F., Imran, M., Iqbal, M., Mirza, O., and Rhaman, M. (2016). Activity of the human rhinovirus 3C protease studied in various buffers, additives and detergents solutions for recombinant protein production. PLoS One *11*, e0153436.

Vanotti, E., Amici, R., Bargiotti, A., Berthelsen, J., Bosotti, R., Ciavolella, A., Cirla, A., Cristiani, C., D'Alessio, R., Forte, B., et al. (2008). Cdc7 kinase inhibitors: pyrrolopyridinones as potential antitumor agents. 1. Synthesis and structure-activity relationships. J. Med. Chem. *51*, 487–501.

Wan, L., Niu, H., Futcher, B., Zhang, C., Shokat, K.M., Boulton, S.J., and Hollingsworth, N.M. (2008). Cdc28-Clb5 (CDK-S) and Cdc7-Dbf4 (DDK) collaborate to initiate meiotic recombination in yeast. Genes Dev. *22*, 386–397.

Weinreich, M., and Stillman, B. (1999). Cdc7p-Dbf4p kinase binds to chromatin during S phase and is regulated by both the APC and the RAD53 checkpoint pathway. EMBO J. *18*, 5334–5346.

Williams, C.J., Headd, J.J., Moriarty, N.W., Prisant, M.G., Videau, L.L., Deis, L.N., Verma, V., Keedy, D.A., Hintze, B.J., Chen, V.B., et al. (2018). MolProbity: more and better reference data for improved all-atom structure validation. Protein Sci. *27*, 293–315.

Winter, G., Lobley, C.M., and Prince, S.M. (2013). Decision making in xia2. Acta Crystallogr. D Biol. Crystallogr. *69*, 1260–1273.

Yamada, M., Watanabe, K., Mistrik, M., Vesela, E., Protivankova, I., Mailand, N., Lee, M., Masai, H., Lukas, J., and Bartek, J. (2013). ATR-Chk1-APC/C-Cdh1-dependent stabilization of Cdc7-ASK (Dbf4) kinase is required for DNA lesion bypass under replication stress. Genes Dev. *27*, 2459–2472.

Yang, J., Cron, P., Thompson, V., Good, V.M., Hess, D., Hemmings, B.A., and Barford, D. (2002). Molecular mechanism for the regulation of protein kinase B/Akt by hydrophobic motif phosphorylation. Mol. Cell *9*, 1227–1240.

Zhuang, L., Yang, Z., and Meng, Z. (2018). Upregulation of BUB1B, CCNB1, CDC7, CDC20, and MCM3 in tumor tissues predicted worse overall survival and disease-free survival in hepatocellular carcinoma patients. Biomed. Res. Int. *2018*, 7897346.

Zhuo, S., Clemens, J.C., Hakes, D.J., Barford, D., and Dixon, J.E. (1993). Expression, purification, crystallization, and biochemical characterization of a recombinant protein phosphatase. J. Biol. Chem. *268*, 17754–17761.

STAR★METHODS

KEY RESOURCES TABLE

REAGENT or RESOURCE	SOURCE	IDENTIFIER
Bacterial and Virus Strains		
BL21(DE3)	Novagen	Cat# 69450
Rosetta 2(DE3)	Novagen	Cat# 71400
Chemicals, Peptides, and Recombinant Proteins		
λ phage protein phosphatase (λPPase), His$_6$-tagged	This work	N/A
HRV14 3C protease, His$_6$SUMO-tagged	This work	N/A
CDC7-DBF4 heterodimers (WT and mutants)	This work and Hughes et al. (2012)	N/A
ATPγS-S40-MCM2(33-47) bisubstrate peptide	This work	N/A
biotinylated MCM2(35-47) peptides (WT and mutants)	This work	N/A
ATPγS	Merck	Cat# 119120
Rink Amide resin	Merck	Cat# 855120
Fmoc-Dpr(Mtt)-OH	Merck	Cat# 852089
Critical Commercial Assays		
SAM2 biotin-capture membrane assay	Promega	Cat# V2861
Deposited Data		
Initial model used for molecular replacement	Hughes et al. (2012)	PDB: 4F9C
Structure of a pCDK2/CyclinA transition-state mimic	Bao et al. (2011)	PDB: 3QHW
CDC7-DBF4 heterodimer in complex with XL413	This work	PDB: 6YA6
CDC7-DBF4 bound to ADP-BeF_3^-	This work	PDB: 6YA7
CDC7-DBF4 bound to bisubstrate peptide	This work	PDB: 6YA8
Oligonucleotides		
Forward primer λPPase 5′-TGCGCTATTACGAAAAAATTGATGG	Sigma-Aldrich	N/A
Reverse primer λPPase 5′-GCGCGTCGACTGCGCCTTCTC CCTGTACC	Sigma-Aldrich	N/A
Forward primer HRV14 3C 5′-GGACGAATTCGGACCAAACACAGA ATTTGCAC	Sigma-Aldrich	N/A
Reverse primer HRV14 3C 5′-GGAGCCTCGAGTTATTGTTTCTCTAC AAAATATTG	Sigma-Aldrich	N/A
Recombinant DNA		
pET-λPPase-His6	This work	N/A
pETSUMO-3C	This work	N/A
pRSFDuet1	Novagen	Cat# 71341
pCDFDuet1	Novagen	Cat# 71340
pRSFDuet-CDC7 (WT and mutants)	This work and Hughes et al. (2012)<	N/A

(*Continued on next page*)

Continued

REAGENT or RESOURCE	SOURCE	IDENTIFIER
Software and Algorithms		
XDS	Kabsch (2010)	http://xds.mpimf-heidelberg.mpg.de/
SCALA	Evans (2006)	http://www.ccp4.ac.uk/html/scala.html
Xia2	Winter et al. (2013)	https://xia2.github.io/
Phaser	McCoy et al. (2007)	http://www.ccp4.ac.uk/html/phaser.html
Phenix Autobuild	Terwilliger et al. (2008)	https://www.phenix-online.org/
phenix.refine	Adams et al. (2010)	https://www.phenix-online.org/
Refmac	Murshudov et al. (1997)	http://www.ccp4.ac.uk/html/refmac5.html
Coot	Emsley and Cowtan (2004)	https://www2.mrc-lmb.cam.ac.uk/personal/pemsley/coot/
MolProbity	Williams et al. (2018)	http://molprobity.biochem.duke.edu/
PyMOL	Warren L. DeLano and Schrödinger LLC	https://pymol.org

RESOURCE AVAILABILITY

Lead Contact

Further information and requests for resources and reagents should be directed to and will be fulfilled by the Lead Contact, Peter Cherepanov (peter.cherepanov@crick.ac.uk).

Materials Availability

Plasmids generated in this study are available upon request.

Data and Code Availability

The coordinates and structure factors were deposited in the Protein Data Bank (PDB) under accession codes 6YA6 (with XL413), 6YA8 (with ADP-BeF_3^-), and 6YA7 (with bisubstrate).

EXPERIMENTAL MODEL AND SUBJECT DETAILS

Recombinant proteins were produced in *E. coli* cells as outlined in Method Details.

METHOD DETAILS

DNA Constructs

For expression in bacteria, DNA fragments encoding deletion mutants of CDC7 were cloned between *Nco*I and *Xho*I sites of pRSFDuet1 (Novagen) for tag-less expression. A DNA fragment encoding residues 210–350 of human DBF4, extended to include a rhinovirus 3C protease cleavage site (LEVLFQGP), was inserted between the *Eco*RI and *Xho*I sites of pCDFDuet1(Novagen) in frame with the vector sequence encoding a hexahistidine (His_6) tag. DNA fragment encoding λ phage protein phosphatase (Zhuo et al., 1993) was PCR-amplified using primers 5′-TGCGCTATTACGAAAAA ATTGATGG and 5′-GCGCGTCGACTGCGCCTTCTCCCTG TACC and λ phage *Hin*dIII digest DNA ladder (New England Biolabs) as template and cloned between *Nde*I and *Sal*I sites of pET20b(+) vector (Novagen), resulting in pET-λPPase-His6. A DNA fragment encoding human rhinovirus 14 (HRV14) 3C protease (Cordingley et al., 1990; Ullah et al., 2016) amplified using primers 5′-GGACGAATTCGGA CCAAACACAGAATTTGCAC and 5′-GGAGCCTCGAGTTATTGTTTCTCTACAAAATATTG was digested with *Eco*RI and *Xho*I for ligation into pET28-SUMO (gift from A.L.B. Ambrosio, University of Sao Paulo, Brazil) to obtain pETSUMO-3C.

Recombinant Proteins

His_6-tagged λ phage phosphatase and His_6-SUMO-tagged HRV14 3C protease were produced in BL21(DE3) cells transformed with pET-λPPase-His_6 or pETSUMO-3C, respectively, by induction with 300 μM isopropyl-β-D-thiogalactoside (IPTG) in Luria broth (LB) at 25°C. Cells were disrupted by sonication in core buffer (500 mM NaCl, 50 mM Tris-HCl, pH 7.5), and the extracts were clarified by centrifugation. His_6-tagged proteins were captured by incubation with NiNTA slurry (Qiagen) in the presence of 20 mM imidazole, and the resin was extensively washed with core buffer supplemented with 20 mM imidazole. The proteins, eluted with 200 mM imidazole in core buffer, were supplemented with 10 mM dithiothreitol (DTT). Phosphatase was dialyzed against cold 50 mM NaCl, 2 mM ethylenediaminetetraacetic acid (EDTA), 0.5 mM DTT, 25 mM Tris-HCl, pH 7.5. After 4 h, buffer was exchanged to 50 mM NaCl, 0.1 mM $MnCl_2$, 25 mM Tris-HCl pH 7.5, and the dialysis continued for additional 4-6 h, at 4°C. His_6-SUMO-tagged HRV14 3C protease was

purified by size exclusion chromatography through a Superdex 200 16/600 column (GE Healthcare) in 200 mM NaCl, 50 mM Tris-HCl, pH 7.5, supplemented with 10 mM DTT and concentrated using a 10-kDa cutoff VivaSpin-20 device. The proteins (10-20 mg/ml) were supplemented with 10% glycerol, snap-frozen in liquid nitrogen and stored at -80°C in small aliquots.

Production and purification of CDC7-DBF4 complexes was carried out as described previously (Hughes et al., 2012), with addition of the dephosphorylation step downstream of His_6 tag affinity chromatography. Rosetta 2 (DE3) cells co-transformed with compatible CDC7 and DBF4 expression constructs were grown in LB containing 50 μg/ml kanamycin and 100 μg/ml spectinomycin at 30°C. At an A_{600} of 0.9–1.0, the culture was supplemented with 50 μM $ZnCl_2$ and 300 μM IPTG. Following overnight induction at 18°C, cells were harvested by centrifugation and stored at -80°C. Cells, thawed in kinase core buffer (50 mM NaH_2PO_4, 300 mM NaCl, 10% glycerol, pH 7.5) supplemented with 0.1 mM PMSF, complete EDTA free protease inhibitor mix, 1 mg/ml lysozyme and 0.5% NP40, were lysed by sonication on ice. The extracts, clarified by centrifugation, were supplemented with 20 mM imidazole and incubated with 3 ml Ni-NTA agarose (Qiagen) for 30 min at 4°C with gentle rocking. The resin was washed with 4 changes of 30 ml kinase core buffer supplemented with 20 mM imidazole. Hexahistidine-tagged CDC7-DBF4 heterodimers were eluted in the presence of 200 mM imidazole; fractions enriched in CDC7-DBF4 heterodimers were pooled, diluted with salt-free buffer to adjust NaCl concentration to 200 mM, supplemented with His_6-tagged λ phage protein phosphatase (1 mg per 15 mg of protein), His_6-SUMO-tagged HRV14 3C protease (1 mg per 50 mg of protein), 1 mM $MgCl_2$, 2 mM $MnCl_2$, and 1 mM DTT and incubated overnight at 4°C. Precipitates were removed by centrifugation, and soluble material was dialysed against a large excess of 50 mM Tris-HCl pH 7.5, 100 mM NaCl, 20 mM imidazole, 2 mM DTT for 2-3 h, at 4°C. Dialysed protein was filtered through a 1-ml HisTrap FF column (GE Healthcare) to remove phosphatase and protease. CDC7-DBF4 heterodimers, collected in the flow through, were diluted 1:5 in salt-free buffer to adjust NaCl concentration to ~80 mM and passed through a 1-ml HiTrap Q HP column (GE Healthcare). CDC7-DBF4 heterodimers, collected in the flow-through, were supplemented with 2 mM DTT and concentrated to ~5 ml using a 10-kDa cut-off VivaSpin-20 device (Sartorius). Recombinant proteins were then further purified by size exclusion chromatography through a Superdex 200 16/600 column in 150 mM NaCl, 25 mM Tris-HCl, pH 7.5. Fractions enriched in CDC7-DBF4 heterodimers were pooled and dialyzed against excess 150 mM NaCl, 75 μM $ZnCl_2$ 2 mM DTT, 25 mM Tris-HCl pH 7.5 overnight at 4°C. The purified heterodimers were concentrated to ~10 mg/ml for immediate use in crystallography or supplemented with 10% glycerol and snap-frozen in liquid nitrogen for later use in kinase assays.

Peptides and Bisubstrates

Peptides were assembled with C-terminal amides following the standard Fmoc chemistry using a 433A instrument (Applied Biosystems) on Rink Amide resin (Sigma-Aldrich). To allow conjugation to ATPγS, the target Ser residue was replaced with aminoalanine using the derivative N-α-Fmoc-N-β-4-methyltrityl-L-diaminopropionic acid. After chain assembly, the side chain methyltrityl group was selectively removed by treatment with 1% trifluoroacetic acid (TFA), 4% triisopropylsilane in dichloromethane. A bromoacetyl moiety was linked to the aminoalanine by reacting the peptidyl-resin with 8.3 eq of bromoacetic acid, 8.3 eq of diisopropylcarbodiimide in a minimum amount of dimethylformamide under stirring condition for 2 h, at ambient temperature. Peptides, released from the resin by treatment with 95% TFA, 2.5% triisopropylsilane, 2.5% water for 2 h, were precipitated with diethyl ether, re-dissolved in water and lyophilized. Peptides were purified by reverse-phase HPLC using 1% acetonitrile, 0.08% TFA in water (buffer A) and 90% acetonitrile, 0.08% TFA in water (buffer B). The ATPγS-peptide conjugates were obtained through a thioether bond formation by reacting the sulfhydryl group of ATPγS and the bromoacetyl moiety. In reported methods (Parang et al., 2001), the purified bromoacetylated peptide was dissolved in a solution methanol:water (4:1) and treated with ATPγS for 24 h at room temperature. For peptides used in this study, triethylammonium bicarbonate buffer (1 M, pH 8.4-8.6) was used to perform this reaction since the bromoacetylated peptides were not soluble in the methanol:water (4:1) solution. Identities and purities of the final products was verified using in-line liquid chromatography mass spectrometry in negative ion mode. Several ATPγS-peptide conjugates were produced, and crystallisation was successful with ATPγS-S40-MCM2(33-47) bisubstrate, containing modified MCM2(33-47) peptide ^{33}RRTDALTZ[pS]PGRDLP^{47}, where Z and [pS] stand for amino alanine and phosphoserine, respectively. The former, replacing natural Ser40 of MCM2, was covalently linked to ATPγS as described above.

In Vitro Kinase Activity Assays

CDC7 kinase assays were carried out essentially as described (Hughes et al., 2012). The substrate peptide spanning residues 35-47 of human MCM2 (^{35}TDALTS[pS]PGRDLP^{47}) was synthetized with a phosphate group attached to Ser41 ([pS]) and N-terminally biotinylated. Each 25-μl kinase reaction contained 5 μg biotinylated substrate, 2.8 nM CDC7-DBF4 heterodimer and 3 μCi of [γ-^{32}P]ATP (3,000 Ci/mmol) in 10 mM $MgSO_4$, 2 mM DTT, 1 mM β-glycerophosphate, 1 mM NaF, 80 μg/ml bovine serum albumin (BSA), 0.1% Nonidet P-40, 0.1 mM ATP, and 40 mM 4-(2-hydroxyethyl)-1-piperazineethanesulfonic acid-NaOH, pH 7.4. Reactions were allowed to proceed for 30 min at 30°C and were terminated by addition of guanidine hydrochloride to a final concentration of 2.5 M prior to spotting onto SAM2 biotin-capture membranes (Promega). The membranes were washed sequentially with 2 M NaCl (three times), 2 M NaCl in phosphate buffered saline (PBS, four times), distilled water (twice), and 95% ethanol. Air-dried membranes were exposed to a phosphor storage screen, and incorporated ^{32}P was quantified using a Storm-860 scanner (GE Healthcare).

Crystallisation, X-Ray Data Collection and Structure Refinement

Crystals were grown in hanging drops by mixing 1 μl CDC7(Δ1-36/Δ228-345/Δ467-533)-DBF4^{MC} heterodimer (10 mg/ml, supplemented with ligands as required) with 1 μl reservoir solution and equilibrating against optimized reservoir solution by vapour diffusion

at 18°C. To crystallise the complex with XL413, the heterodimer supplemented with 5 mM inhibitor was mixed with and equilibrated against 21% polyethylene glycol (PEG) 1500, 5 mM $MgCl_2$, and 0.1 M MIB (sodium malonate/imidazole/boric acid buffer system, Molecular Dimensions), pH 5.5. The crystals were cryoprotected by soaking in 5-μl drops containing 2.5 mM XL413, 100 mM NaCl, 2.5 mM $MgCl_2$, 10 mM Tris-HCl and 80 mM MIB, pH 5.5, with stepwise increases in PEG1500 (in 1% steps) and PEG400 (5% steps) to the final concentrations of 24% and 20%, respectively.

Microseeding using crushed crystals of the kinase-XL413 complex was used to nucleate the remaining two crystal forms of the CDC7(Δ1-36/Δ228-345/Δ467-533)-DBF4^{MC} heterodimer described in this work. Crystals of the heterodimer in the presence of 2 mM ADP and 2 mM BeF_3 were grown using reservoir solution containing 21% PEG1500, 15% v/v acetonitrile, 20 mM $MgCl_2$, and 0.1 M MMT (DL-malic acid/2-N-morpholinoethanesulfonic acid/Tris buffer system, Molecular Dimensions), pH 6.57. The crystals were cryoprotected by soaking in 5-μl drops containing 75 mM NaCl, 20 mM $MgCl_2$, 1 mM ADP, 5 mM Tris-HCl, 70 mM MMT, pH 6.57, 15% acetonitrile, 1 mM BeF_3 with stepwise increases in PEG1500 (in 1% steps) and MPD (5% steps); the optimal cryogenic condition was achieved with a combination of 22% PEG1500 and 20% MPD.

Crystals of CDC7(Δ1-36/Δ228-345/Δ467-533)-DBF4^{MC} in complex with ATPγS-S40-MCM2(33-47) bisubstrate were likewise nucleated by streak seeding and grown in 18% PEG1500, 8% MPD in 0.1 M PCTP (sodium propionate/sodium cacodylate trihydrate/Bis-Tris propane buffer system, Molecular Dimensions), pH 6.5. For cryoprotection, crystals were incubated in 5-μl drops containing 1 mM ATPγS-S40-MCM2(33-47), 75 mM NaCl and 80 mM PCTP, pH 6.5 with stepwise increases in PEG1500 (in 1% steps) and MPD (5% steps) to the final concentrations of 19% and 20%, respectively.

Cryoprotected crystals were snap-frozen by plunging in liquid nitrogen. X-ray diffraction data on the crystals of the CDC7(Δ1-36/Δ228-345/Δ467-533)-DBF4^{MC} heterodimer complex in complex with XL413 were collected on beamline BM14 of the European Synchrotron Radiation Facility (ESRF, Grenoble, France). Diffraction of the remaining two crystal forms was measured on beamline I03 of the Diamond Light Source (Oxfordshire, UK). The data were integrated and merged using XDS (Kabsch, 2010) and Scala (Evans, 2006) via Xia2 automatic pipeline (Winter et al., 2013). The structures were solved by molecular replacement in Phaser (McCoy et al., 2007), using the previously reported structure (PDB: 4F9C) (Hughes et al., 2012) as a search model; the extended KI-2 was built using Phenix Autobuild (Terwilliger et al., 2008). The models were iteratively improved by manual building into *Fo-Fc* difference maps in Coot (Emsley and Cowtan, 2004) and refinement in Refmac (Murshudov et al., 1997) and/or Phenix refine (Adams et al., 2010). Quality of the refined models was evaluated using MolProbity (Williams et al., 2018). X-ray data reduction and final model quality statistics are given in Table 1. Protein structure images were generated using PyMOL software (https://pymol.org).

QUANTIFICATION AND STATISTICAL ANALYSIS

Statistics generated from X-ray data processing, model refinement and validation are shown in Table S1. *In vitro* kinase assays were carried out in triplicate, all standard deviations were calculated using n-1 method, as implemented in Excel (Microsoft).

Structure, Volume *28*

Supplemental Information

Structural Basis for the Activation and Target Site Specificity of CDC7 Kinase

Samual D. Dick, Stefania Federico, Siobhan M. Hughes, Valerie E. Pye, Nicola O'Reilly, and Peter Cherepanov

Figure S1

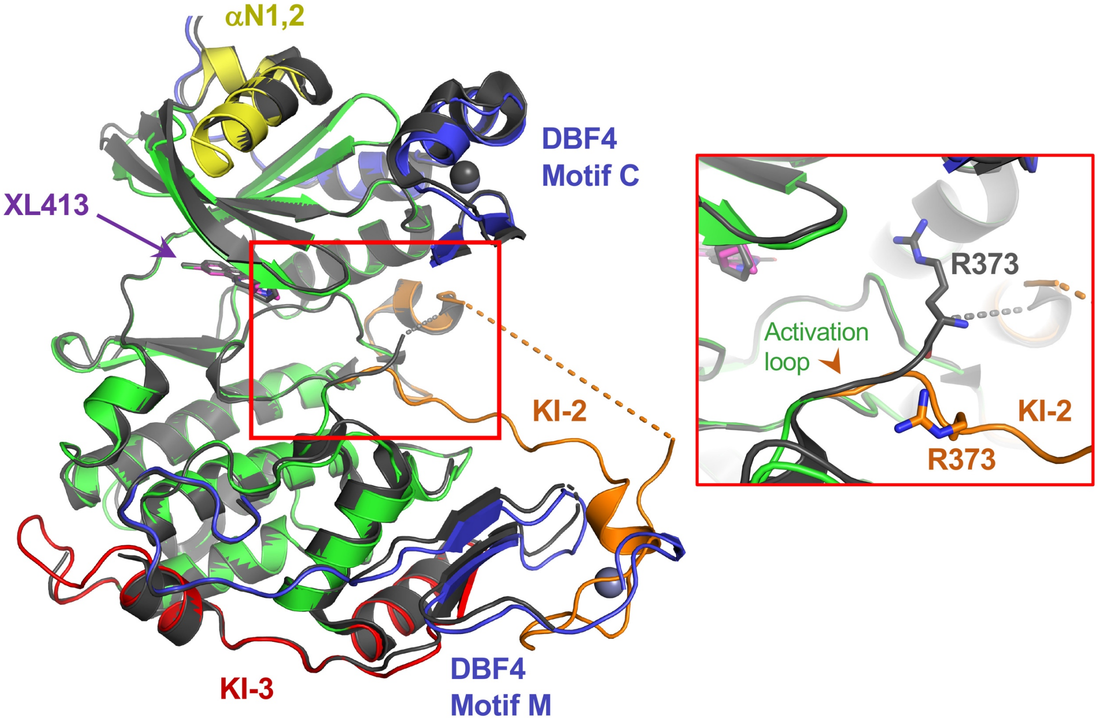

Figure S1. Comparison of the CDC7-DBF4 constructs with Δ228-359 (PDB ID 4F9C, grey) and Δ228-345 (this work, colored as in Fig. 2A) deletions in KI-2. Related to Figure 2A. Protein and the bound inhibitor (XL413) are depicted as cartoons and sticks, respectively. A zoomed view of the CDC7 activation segment region is shown in inset; note that the more extensive deletion in KI-2 leads to a disorder of the CDC7 activation segment.

Figure S2

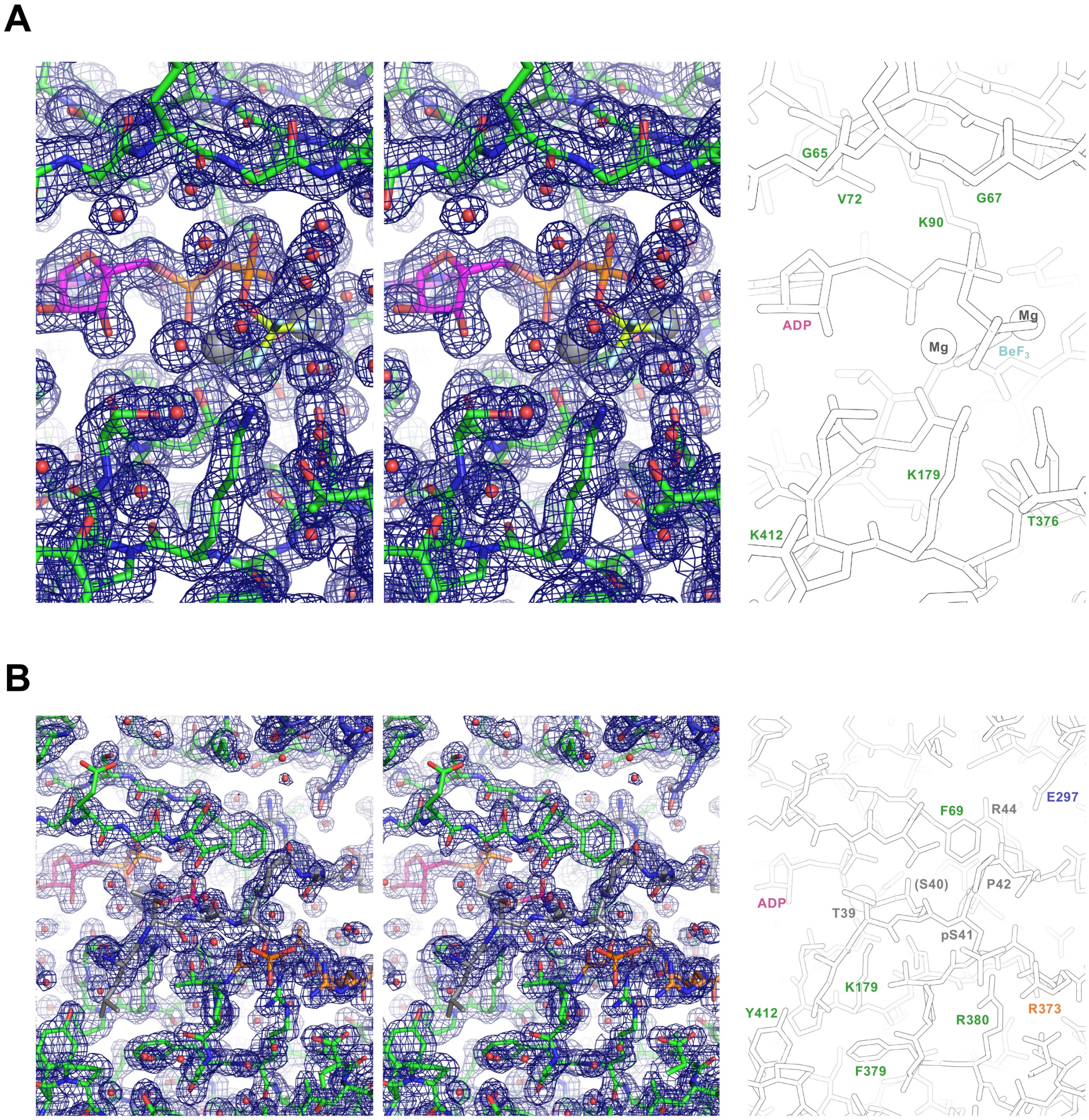

Figure S2. Stereo views of the final electron density maps of ADP-BeF3$^-$ (A) and bisubstrate (B) complexes. Related to Figures 2C and 3A. Weighted *2Fo-Fc* maps, contoured at 1 σ, are shown as blue mesh. Protein residues and ligands are depicted as sticks, water molecules as small red spheres and metal ions as grey spheres. Nucleotides and selected amino acid residues are indicated on the black and white images to the right.
